# Coordinate up-regulation of *TMEM97 *and cholesterol biosynthesis genes in normal ovarian surface epithelial cells treated with progesterone: implications for pathogenesis of ovarian cancer

**DOI:** 10.1186/1471-2407-7-223

**Published:** 2007-12-11

**Authors:** Cathy B Wilcox, Grace O Feddes, Joan E Willett-Brozick, Lih-Ching Hsu, Julie A DeLoia, Bora E Baysal

**Affiliations:** 1 Department of Obstetrics, Gynecology and Reproductive Sciences, University of Pittsburgh School of Medicine, Pittsburgh, PA 15213, USA; 2 Department of Pathology Yale University School of Medicine 310 Cedar Street, BML B38 New Haven, CT 06520-8023, USA

## Abstract

**Background:**

Ovarian cancer (OvCa) most often derives from ovarian surface epithelial (OSE) cells. Several lines of evidence strongly suggest that increased exposure to progesterone (P4) protects women against developing OvCa. However, the underlying mechanisms of this protection are incompletely understood.

**Methods:**

To determine downstream gene targets of P4, we established short term *in vitro *cultures of non-neoplastic OSE cells from six subjects, exposed the cells to P4 (10^-6 ^M) for five days and performed transcriptional profiling with oligonucleotide microarrays containing over 22,000 transcripts.

**Results:**

We identified concordant but modest gene expression changes in cholesterol/lipid homeostasis genes in three of six samples (responders), whereas the other three samples (non-responders) showed no expressional response to P4. The most up-regulated gene was *TMEM97 *which encodes a transmembrane protein of unknown function (MAC30). Analyses of outlier transcripts, whose expression levels changed most significantly upon P4 exposure, uncovered coordinate up-regulation of 14 cholesterol biosynthesis enzymes, insulin-induced gene 1, low density lipoprotein receptor, *ABCG1*, endothelial lipase, stearoyl- CoA and fatty acid desaturases, long-chain fatty-acyl elongase, and down-regulation of steroidogenic acute regulatory protein and *ABCC6*. Highly correlated tissue-specific expression patterns of *TMEM97 *and the cholesterol biosynthesis genes were confirmed by analysis of the GNF Atlas 2 universal gene expression database. Real-time quantitative RT-PCR analyses revealed 2.4-fold suppression of the *TMEM97 *gene expression in short-term cultures of OvCa relative to the normal OSE cells.

**Conclusion:**

These findings suggest that a co-regulated transcript network of cholesterol/lipid homeostasis genes and *TMEM97 *are downstream targets of P4 in normal OSE cells and that *TMEM97 *plays a role in cholesterol and lipid metabolism. The P4-induced alterations in cholesterol and lipid metabolism in OSE cells might play a role in conferring protection against OvCa.

## Background

Ovarian cancer (OvCa), with a lifetime incidence of approximately 1%, accounts for more deaths than all other gynecologic malignancies combined [1]. Approximately 90% of OvCas originate from the ovarian surface epithelium (OSE), a single layer of cuboidal cells covering the ovaries [2]. Although many somatic gene defects have been detected in OvCa, genetic alterations unique to OvCa have been difficult to identify. Consequently, the molecular mechanisms leading to OvCa remain amongst the least understood of common cancers. Certain epidemiological variables such as advancing age, low parity, infertility, and family history are associated with increased risk; whereas oral contraceptive use is associated with decreased risk of OvCa [3]. Several biological models have been advanced to explain the mechanisms of these risk-modifying factors. The incessant ovulation hypothesis postulates that repetitive wounding and healing of the ovarian surface where the OSE cells proliferate to repair the rupture during ovulation predisposes to OvCa by leading to accumulation of mutations [4]. The gonadotropin theory postulates that increased levels of pituitary gonadotropins during ovulation and sustained high levels during menopause stimulate production of estrogens and other hormones to increase risk of OvCa. Although incessant ovulation or chronic gonadotropin stimulation could contribute to the etiopathogenesis of OvCa, it appears that other hormonal factors such as androgenic and progestogenic stimulations also play important roles [5].

Risch [5] first proposed a protective role for progesterone (P4) against OvCa on the bases of multiple lines of evidence. First, the protective effect of pregnancies, and especially of twin pregnancies, against OvCa has been attributed to the elevated levels of P4 in addition to the suppression of ovulation [5], because the degree of protection conferred by pregnancies seems too high to be explained simply by the pause in ovulation. Second, P4 reduces the proliferation rate in normal OSE cells both in a primate model and in tissue culture [6-8] and suppresses the transformed phenotype *in vitro *[9]. Third, P4 is a potent inhibitor of proliferation in cultured human OSE cells at concentrations similar to the levels reached during pregnancy [10]. One potential mechanism for this protective effect is that high does of P4 could reduce invasiveness of OvCa by reducing epithelial membrane fluidity [11]. Accordingly, it has been observed that pretreatment of mice with P4 reduced the numbers of OvCa implants in the abdominal cavity, whereas P4 treatment had no effects once the tumors were implanted [12].

Despite evidence for an anti-carcinogenic role for P4 in OvCa, it has been difficult to fully understand the underlying mechanisms. The intracellular effects of P4 are mediated primarily by intracellular P4 receptors (PR) that are expressed as two protein isoforms, PR-A and PR-B, encoded by the same genetic locus [13]. The implicated mechanisms underlying the protective effects of P4 against OvCa include induction of cell cycle arrest or apoptosis, possibly through activation of the extrinsic apoptotic pathway and Fas/FasL signaling, alternative expression of transforming growth factor beta isoforms, and alterations of the fluid dynamics of plasma membranes in OvCa cells [14]. In a recent study, that analyzed ~2400 genes in OvCa lines, Syed *et al.*[15] found four suppressed genes that were derepressed upon P4 exposure. Depression of these genes suppressed the transformed phenotype in OvCa cells. Although these studies have provided clues for potential mechanisms of P4's anti-carcinogenic effects on OvCa cells, little is known about their relevance for P4's prophylactic role against OvCa. In other terms, mechanisms regulated by P4 that could prevent the neoplastic transformation of normal OSE cells are unknown.

To the best of our knowledge, no study has systematically addressed the transcriptional impact of P4 on normal OSE cells. In the light of strong epidemiological evidence implicating P4 as a protective hormone against OvCa, it is plausible that certain downstream effectors of P4 in normal OSE cells could be involved in protection against OvCa. Here, we conducted a global survey of the expression changes induced by high concentrations of P4 exposure for five days on normal OSE cells obtained from six women. Our analysis, using Affymetrix oligonucleotide microarray chips, revealed a coordinate and highly significant up-regulation of multiple genes in the cholesterol and fatty acid biosynthesis pathways in response to P4.

## Methods

### Subjects and cell culture

Primary cultures of OSE cells were obtained following informed consent from cases who underwent hysterectomy and oophorectomy for various clinical indications other than OvCa. In all cases the ovaries showed benign alterations upon postoperative histological examination (Table 1). Ovarian cancer samples were obtained from Magee-Womens Hospital Tissue Bank. The research protocols were approved by the University of Pittsburgh IRB review committee.

**Table 1:** Clinical information for patients providing ovarian surface epithelium

Case	Age	Reason for surgery	Final Diagnosis
1	45	Fibroid uterus	Serosal adhesions and epithelial inclusions
2	74	Pelvic mass	Serous cystadenofibroma
3	49	Prophylactic oopherectomy for sister had early-onset (38 y) ovarian cancer	Hemorrhagic follicular cysts
4	44	Menorrhagia and fibroids	Follicular cysts
5	76	Endometrial cancer	Epithelial inclusions
6	43	Pelvic mass	Epithelial inclusions

**Figure 1:**
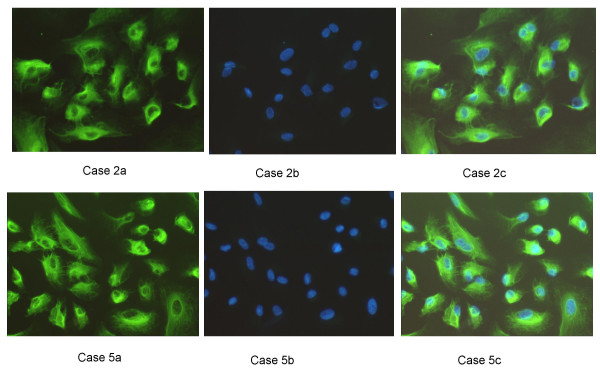
**Ovarian surface epithelial (OSE) cells in culture**. The epithelial origin of primary OSE cultures is demonstrated for one responder (case 2) and one non-responder cultures (case 5). a, cytokeratin staining, b, DAPI counterstain, c, merge of cytokeratin and DAPI staining. Magnifications: 10 × 20. The majority of OSE cultures expressed keratin, confirming their epithelial origin.

The OSE cell cultures were established by brushing the epithelial lining of the fresh ovaries. The epithelial content of the OSE cells were confirmed by immunofluorescence staining (Figure 1). Briefly, primary OSE cells were spun onto slides using a cytospin centrifuge and fixed in cold methanol at -20°C, and subjected to immunofluorescence staining as described previously [16]. A mouse monoclonal anti-cytokeratin antibody (clone K8.13, 1:500 dilution, ICN, Irvine, CA) was used for cytokeratin staining. Secondary antibody used was FITC-conjugated goat anti-mouse IgG (Southern Biotechnology Associates, Birmingham, AL). The nucleus was counterstained with 4',6'-diamidino-2-phenylindole (DAPI). The purity of the epithelial component in each OSE cell culture for the six cases were 100%, 100%, >90%, 90%, 100% and >80%, respectively.

For P4 exposures, OSE cells were grown in Dulbecco's Modified Eagle's Medium (DMEM) supplemented with 15% fetal bovine serum (FBS) and streptomycin/penicillin. 48 hours before the hormone treatment, the OSE cells were plated in four T25 flasks, the medium replaced with DMEM supplemented with 15% charcoal-stripped FBS and the cells allowed to reach 70–75% confluence. The medium was then replaced with fresh media containing 10^-6 ^M P4 (Sigma) or vehicle (ethanol). The final concentration of ethanol in the media was 0.03% both in the P4 and control experiments. The hormone-treated cells were exposed to P4 for a total of 5 days, with three fresh additions of media containing P4 (or vehicle) every 48 hours for stable bioavailability as described [10]. At the end of the fifth day, the medium was removed. The cells were washed with PBS, collected following trypsin treatment and immediately frozen for RNA extraction. Cells from duplicate flasks were combined. To test bioavailability of P4 in tissue culture, we used breast cancer cell line MCF-7 to confirm upregulation of human DLG5 gene by P4 [17] and found 5.2-fold and 2.2-fold gene expression increases by real-time RT-PCR at 8 and 24 hours, respectively.

### RNA extraction

The total RNAs were extracted following a commercial protocol (RNA-Bee, Tel-Test, Inc., Friendswood, TX) that used phenol and guanidine thiocyanate. The total RNAs were resuspended in DEPC-treated distilled water and further purified using RNeasy mini-columns (Qiagen, Valencia, CA) in preparation for microarray analyses. After column purification, the total RNAs were quantitated by absorbance at 260 nm using a Beckman DU-64 spectrophotometer. Approximately 10 μg of total RNA were provided for microarray analysis of global gene expression patterns. After the RNeasy™ purification step and demonstrating an OD 260/280 ratio of 1.8 or higher, The University of Pittsburgh Microarray facility confirmed RNA integrity via Agilent Bioanalyzer 2100.

### Microarray hybridization and data processing

The high-density microarrays (Affymetrix gene chip type U133A) used in this study contained 22,283 unique human transcripts derived from the RefSeq database and were commercially available from Affymetrix, Santa Clara, CA. The hybridizations to microarrays were performed by The University of Pittsburgh Microarray Facility. All RNAs passed quality control tests before they were processed further for microarray hybridizations. Afterwards, approximately 5 μg of total RNA was used to generate double-stranded cDNA by reverse transcription using a cDNA synthesis kit (Superscript Choice System; Life Technologies, Inc., Rockville, MD) that used an oligo(dT)_24 _primer containing a T7 RNA polymerase promoter 3' to the poly T (Geneset, La Jolla, CA), followed by second-strand synthesis. Labeled cRNA was prepared from the double-stranded cDNA by *in vitro *transcription by T7 RNA polymerase in the presence of biotin-11-CTP and biotin-16-UTP (Enzo, Farmington, NY). The labeled cRNA was purified over RNeasy columns and hybridized to gene chips following the manufacturer's protocol. cRNAs derived from the control unexposed cells and the P4 exposed cells were individually hybridized to chips using identical experimental protocols to reduce sources of technical variation.

Each transcript is represented on the chip as a set of 16–20 probe pairs with each pair containing a perfect match and a mismatch. The mismatch is the same oligonucleotide as the perfect match but with a single base substitution at the central position. The amount of non-specific hybridization is corrected by comparing the hybridization level between the perfect match and the mismatch. Affymetrix 418 Array Reader was used to scan the fluorescently tagged microarrays. Global scaling techniques were used for all probe sets, to make the average intensity of each image equal to an arbitrary target intensity, set to 150. The expression data (signal intensities) across the chips were normalized so that the expression levels of a gene were directly comparable across the chips. The normalization of all 12 chips was performed by D-chip software to the probe level in the P4-exposed chip for sample#3, which was arbitrarily chosen as the standard [18]. Results of the genome-wide gene expression microarrays are submitted to the public repository database, GEO: Gene Expression Omnibus [19].

### Quantitative RT-PCR (qRT-PCR) analyses

qRT-PCR was used to validate the up-regulation of genes identified by microarray analysis using an ABI Prism 7700 Sequence Analyzer according to the manufacturer's recommendations (Applied Biosystems, Foster City, CA). The comparative threshold cycle method was used for the calculation of relative transcript amounts as specified by the manufacturer. Complementary DNA was synthesized with SuperScript II reverse transcriptase (RT) using total RNA. qRT-PCR analysis of cDNAs for each gene included a negative control template generated without addition of reverse transcriptase during cDNA synthesis. Average fold changes of target genes were determined relative to the internal control genes. Each target and control gene was amplified in separate wells in triplicates. The control genes were chosen from among the housekeeping genes whose expression levels did not change upon P4 exposure in the microarray data. The probes for qRT-PCR were purchased commercially from Applied Biosystems. PCR efficiency tests were performed before the transcript quantification assays to confirm that the amount of input cDNA was inversely proportional to the threshold cycle number for each gene (i.e. increased cDNA input reduced the detection threshold in a linear fashion).

### Expression and Genotyping of progesterone receptor (PR) Gene

PR gene polymorphisms are screened by standard PCR amplification and restriction enzyme (RE) digestion. We screened the V660L polymorphism by PCR amplifying PR gene exon 4 by PCR oligonucleotide primers 5'-TTCTCTGTAGGTCGAAAATTTAAAA-3' and 5'-TATTATTACCTGGCAATGATTTAG-3' at an annealing temperature of 59°C and digesting with the *Bsr*I RE. The allele for leucine destroys the *Bsr*I RE site. Similarly, the +331 G/A SNP in the promoter region was screened by PCR amplification using oligonucleotide primers 5'-CACTACTGGGATCTGAGATC-3' and 5'-TTTATCTCCCGACTTTTTCTC-3' at an annealing temperature of 55°C and *Nla*IV RE digestion. The A allele destroys the *Nla*IV RE site. RE enzymes were purchased from New England Biolabs (NEB). To investigate the expression status of PR, we conducted real-time qRT-PCR analysis using PR primers amplifying the transcript portion common to both the PR-A and PR-B isoforms and specific to the PR-B isoform in the P4-exposed and baseline control cultures in samples 2,3 and 6. This analysis showed that both isoforms of PR were expressed in the responder and non-responders cell cultures without significant changes upon P4 exposure (Additional File 1).

### Statistical analysis

**Figure 2:**
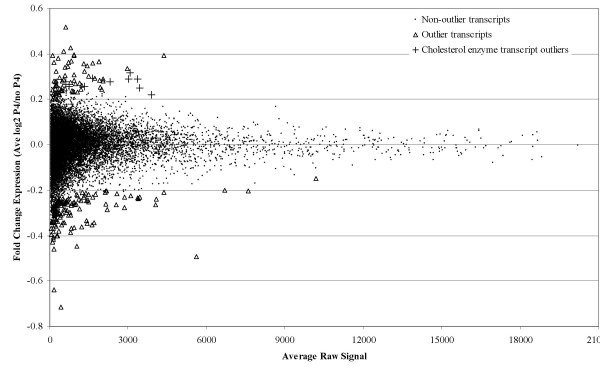
**Genome-wide view of fold-change in gene expression by P4 exposure**. Average of raw signals in 12 microarrays (x-axis) are plotted against the average of log_2_-based fold-changes in expression in 6 microarray-pairs (y-axis) in the presence of 10^-6 ^M P4 for five days. 224 transcripts were statistically determined to be outliers (shown by triangles). Eleven of the outliers encode cholesterol biosynthetic enzymes (shown by plus signs). No transcript showed more than 2-fold average change (i.e., no points are dispersed beyond y = 1 or y = -1).

The normalized expression data from twelve chips were analyzed jointly to detect genes and/or pathways regulated by P4. The signal intensities of the transcripts in the chips provide a quantitative measure of their relative abundance. To assess gene expression changes upon P4 exposure, for each transcript, we calculated signal log ratio (P4/control) in each of the six experimental pairs and plotted the average of the six signal log ratios on the y-axis against the average signal intensity in twelve chips on the x-axis (Figure 2). The signal log ratio is a measure of fold change in expression level for a transcript in the P4-exposed sample relative to its unexposed control. The change is expressed as the log_2 _ratio. A signal log ratio of 1 is the same as a 2-fold increase and signal log ratio of -1 is the same as a 2-fold decrease in signal intensity of a transcript. We assumed that signal intensity of a transcript that is not regulated by P4 would randomly fluctuate around a mean value on repeated measurements. Therefore the average signal log ratios of the six pairs would randomly distribute on the y-axis close to zero. We also assumed that signal intensities of transcripts expressed at similar levels would show similar levels of variations, as strongly suggested in Figure 2, on repeated measurements and therefore their average signal log ratios would also be comparable. However, if a transcript is regulated by P4, its average signal intensity would be displaced towards higher (up-regulation) or lower (down-regulation) mean values on the x-axis and its average signal log ratio would be dispersed further away from zero on the y-axis relative to the other transcripts of similar signal intensity, which are not regulated by P4. We refer to the transcripts with the most extreme average signal log-ratios (on y-axis) compared to those transcripts expressed *at similar levels *as outliers and evaluate them as candidates for regulation by P4.

Different approaches to identify the outlier transcripts are conceivable. For example, distribution of signal log ratios for all transcripts can be approximated by continuous curves that define significance boundaries [20]. Here, we first ranked the transcripts on the basis of their average signal intensities in all 12 chips and generated a group from every consecutive 500 transcripts. The grouping of transcripts with similar expression levels safeguarded against preferential selection of lowly expressed transcripts which showed greater variations in their signal log ratios. As expected, the signal log ratios of all transcripts distributed symmetrically around the x-axis (Figure 2). Then, starting with the most abundantly expressed transcripts, we found the transcripts whose average signal log ratios were most extreme within each group of 500 transcripts. We found the transcripts whose average signal log ratios were located more than 3 standard deviations (SD) away from the mean of the group and whose fold changes were located more than 2 interquartile regions (iqr) away from the first and third quartile of the group. We defined these transcripts as outliers. In a normal distribution, 99% of the data points fall within three SDs of the mean. Whereas the 2iqr limits around the median delimits moderate outliers in any distribution. These two measures of dispersion were jointly used to detect the outlier transcripts at a moderate stringency level even if the distribution of signal-log ratios may not be normal in a group.

The outlier transcripts were then submitted to the Affymetrix web site [21] to identify biological pathways and clusters listed in the Gene Ontology database associated with the outlier transcripts. No particular pathway or cluster was expected to be significantly enriched by the outlier transcripts under the null hypothesis. To test this hypothesis, we conducted one-sided two-by-two Fisher's exact test [22] for enrichment of each of the pathways or clusters by the outliers. The total number of annotated (i.e., assigned to a pathway) outliers and the total number of annotated non-outliers were constant entries in each Fisher's exact test, whereas the numbers of outlier and non-outlier transcripts in a specific biological pathway/cluster were entered as variable values. We implemented two conservative measures to the serial significance testing of pathways for enrichment. First, the number of outlier transcripts belonging to a pathway was reduced by 1, as the first outlier transcript hit to a pathway was designated as the "ascertaining transcript" which could be a chance ascertainment. This step eliminated from further testing all pathways represented by only one transcript. Second, because some pathways were expected to show significant enrichment by chance as a result of multiple testing, we performed a Bonferroni correction and multiplied the nominal p-values by the total number of pathways ascertained by the outliers before declaring significance.

## Results

### Analysis of gene expression changes in OSE cells upon P4 exposure

The OSE cultures derived from six women (Table 1) were exposed to P4 (10^-6 ^M) for five days and gene expression changes were profiled in P4-exposed and baseline control cultures using U133A Affymetrix chips. These exposure conditions were chosen because comparable serum P4 levels (~552 nM) are reached at term in normal pregnancies [23] and because five days of exposure to 10^-6 ^M P4 slows down proliferation in OSE cells *in vitro *[10]. Thus these conditions were likely to mimic the physiological effects of long-term P4 exposure. The average signal intensities obtained from 12 normalized chips plotted against average signal-log ratios of the expressional changes from six experimental pairs are shown for 22, 283 transcripts in Figure 2 (also see methods). Transcripts expressed at lower levels showed more variation than the ones expressed at higher levels (Figure 2). The dependence of signal-log ratios, which is a measure of fold-change, on signal intensity was previously recognized [24], and is attributed to small random changes in signal intensities which could inflate fold-changes in less abundant transcripts than they would do in highly expressed genes. To lessen the impact of this artifact, 853 of the most-weakly expressed transcripts (~3.8% of all transcripts), which fell below an arbitrary signal intensity cutoff level of 50, were removed from further analysis. The remaining 21,430 transcripts were evaluated further for gene expression changes upon P4 exposure.

### Outlier detection and pathway analysis

The average of the signal log ratios in six experimental pairs revealed that no transcript showed a change in gene expression level more than two-fold upon P4 exposure (Figure 2). To determine smaller but statistically significant gene expression changes, we evaluated 224 transcripts (outliers) that showed the greatest fold-changes in gene expression upon P4 exposure relative to the transcripts expressed at similar levels. Next, we tested whether these outlier transcripts were enriched for any of the biological pathways/clusters listed in The Gene Ontology database (see methods). One hundred sixty seven of the outlier transcripts were annotated to a cellular pathway or cluster. These annotated outlier transcripts belonged to 551 biological pathways (Table 2). Although no particular pathway is expected to be enriched by the outlier transcripts (null hypothesis), we found that several metabolic pathways involving lipid biosynthesis were significantly overrepresented by the outlier transcripts (Table 2). The most significant enrichment was found in the cholesterol biosynthesis pathway: eleven of the 36 transcripts in this pathway were present among the outliers, although none (0.4) was expected under the null hypothesis (Figure 2). These eleven transcripts, encoding seven enzymes in the cholesterol biosynthesis pathway, were also responsible for the significant enrichment of other lipid metabolism pathways (Table 2).

**Table 2:** Biologic pathways and clusters enriched by the outlier transcripts

**Gene Ontology Term**	**Annotated transcripts on array**	**Annotated transcripts in outliers (all 6 cases)**	**^a^P-values (6 cases)**	**Annotated transcripts in outliers (3 responder cases)**	**^b^P-values (3 responder cases)**	**^c^Annotated transcripts in outliers (3 non-responder cases)**
**All pathways and clusters**	15 047	167	--	188	--	94
**Lipid metabolism**	754	21	NS	30	2.76 × 10^-4^	4
**Cellular lipid metabolism**	602	18	NS	29	9.65 × 10^-6^	3
**Lipid biosynthesis**	255	17	3.93 × 10^-5^	28	1.33 × 10^-13^	3
**Alcohol metabolism**	324	11	NS	23	3.44 × 10^-7^	0
**Steroid metabolism**	198	12	1.17 × 10^-2^	24	2.36 × 10^-12^	1
**Steroid biosynthesis**	90	12	4.47 × 10^-6^	23	5.58 × 10^-19^	1
**Sterol metabolism**	93	11	7.12 × 10^-5^	21	5.46 × 10^-16^	0
**Sterol biosynthesis**	44	11	3.07 × 10^-8^	20	9.91 × 10^-22^	0
**Cholesterol metabolism**	85	11	3.01 × 10^-5^	19	3.58 × 10^-14^	0
Cholesterol biosynthesis*	36	11	4.13 × 10^-9^	18	3.50 × 10^-20^	0
**Isoprenoid metabolism**	18	5	2.39 × 10^-2^	7	3.73 × 10^-5^	0
**Isoprenoid biosynthesis**	16	5	1.45 × 10^-2^	7	1.65 × 10^-5^	0
**Mitotic cell cycle**	319	4	NS	15	4.61 × 10^-2^	0
**Nuclear division**	268	2	NS	14	2.97 × 10^-2^	0
**M phase of mitotic cell cycle**	213	2	NS	14	2.70 × 10^-3^	0
**Mitosis**	209	2	NS	14	2.20 × 10^-3^	0
**Phosphate transport**	106	8	NS	10	5.30 × 10^-3^	0

^a ^Fisher's exact test, corrected for multiple pathway testing (n = 551).

^b ^Fisher's exact test, corrected for multiple pathway testing (n = 542).

^c ^No significant enrichment for any of the 449 pathways.

NS: Not significant

### Distinct responses of OSE cells to P4

An inspection of the gene expression changes in the eleven outlier transcripts in the cholesterol biosynthesis pathway in each experimental pair revealed that whereas the cultures derived from cases 1, 2 and 4 (responder group) showed consistent up-regulation of these transcripts, the other three cultures (non-responder group) did not show appreciable changes in the transcript levels. This finding prompted us to analyze the outlier transcripts of the two groups separately. The responder group had 18 upregulated outlier transcripts encoding 11 genes in the cholesterol biosynthesis pathway, whereas the non-responder group had no significant enrichment for any biological pathway (Table 2). A full list of outlier transcripts detected in the three responder cases is provided in Additional File 3.

*TMEM97*, encoding a transmembrane protein of unknown function (MAC30), showed the highest fold-change (1.94-fold, Additional File 3) among the upregulated outlier transcripts in the responder group. *TMEM97 *was first identified as a downregulated gene in meningioma [25] and was later found to be transcriptional target of the *BRCA1 *gene [26]. *TMEM97 *is strongly expressed in the pancreas and other gastrointestinal tissues and is downregulated in pancreatic cancer [27].

**Figure 3:**
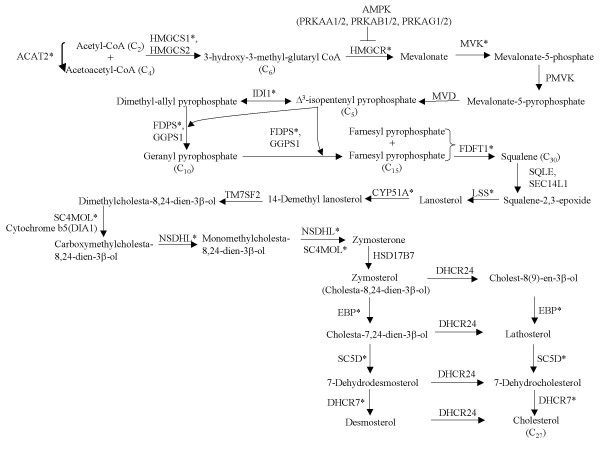
**An overview of the cholesterol biosynthesis pathway**. The enzymes whose transcripts are upregulated upon P4 exposure are shown by asterisks. The full names of the gene symbols are listed in Additional File 2. The cholesterol is synthesized from acetyl-CoA. HMGCR is the rate limiting enzyme because reduction of 3-hydroxy-3-methylglutaryl CoA to mevalonate is the committed step in cholesterol formation. Mevolanate is converted to isopenthenyl pyrophosphate by three sequential reactions requiring ATP. Then squalene is synthesized from six molecules of isopenthenyl pyrophosphate. Then squalene epoxide is cyclized to lanosterol which is converted into cholesterol. The carbon numbers of some intermediates are shown in parenthesis. P4 upregulates transcripts encoding enzymes located throughout the complex biosynthetic pathway. Whereas PRKAA1/2, PRKAB1/2, PRKAG1/2, the genes for subunits of AMP-dependent protein kinase (AMPK), which inhibits HMGCR, appear not to be regulated by P4 (Additional File 2).

The presence of 18 transcripts encoding 11 genes in the cholesterol biosynthesis pathway among the outlier transcripts of the responder group further prompted us to evaluate the expressional changes of other genes in the cholesterol biosynthesis pathway (Figure 3). We found that transcripts encoding lanosterol synthase, sterol-C4-methyl oxidase-like, and sterol C5 desaturase were also among the outliers (Additional File 2) although they were annotated as part of steroid or sterol biosynthesis, but not cholesterol biosynthesis in the Gene Ontology database. Inclusion of these genes brought the total number of upregulated genes in the cholesterol biosynthesis pathway to 14. In addition, transcripts for other cholesterol biosynthesis genes such as squalene epoxidase were also upregulated in the responder group, although they were not detected as outliers (Additional File 2).

The profound impact of P4 on cholesterol metabolism was also evident by transcriptional regulation of other important genes in cholesterol metabolism. Insulin induced gene-1 (INSIG-1), low density lipoprotein receptor (LDLR), and ATP-binding cassette transporter G1 (ABCG1) were upregulated; steroidogenic acute regulatory protein (StAR) and ATP-binding cassette transporter C6 (ABCC6) were downregulated (Additional File 2). In addition, several genes involved in fatty acid metabolism including fatty acid desaturases 1 and 2 (FADS1 and 2), stearoyl-CoA desaturase (SCD), endothelial lipase, phospholipase A2, group IVA, long-chain fatty-acyl elongase, cytochrome P450, subfamily IIC, polypeptide 18 were among the outlier transcripts (Additional File 2).

### Confirmation of transcriptional up-regulation by quantitative RT-PCR (qRT-PCR)

To confirm the transcriptional up-regulation of outlier transcripts in the microarray data, we selected *TMEM97 *and three upregulated genes in the cholesterol biosynthesis pathway: HMG CoA reductase (HMGCR), the rate-limiting enzyme in the cholesterol biosynthesis, isopentenyl-diphosphate delta isomerase (IDI1) and 7-dehydrocholesterol reductase (DHCR7) for additional analyses. We used two genes as controls, small nuclear ribonucleoprotein 70 kDa polypeptide (SNRP70) and ubiquitin carboxyl-terminal esterase L3 (UCHL3), which were expressed at similar levels to the tested genes but whose expressional levels did not change upon P4 exposure in the microarray data. For all four tested genes, the real-time quantitative RT-PCR analysis showed expressional changes similar to the microarray results in two responder (cases 2,4) and one non-responder sample (case 6) (Table 3).

**Table 3:** qRT-PCR confirmation of gene regulation (fold-change) with P4 exposure

Case	HMGCR	DHCR7	IDI1	MAC30	Average fold-change in four genes
2^a^	1.87	1.72	1.98	2.65	2.06
4^a^	1.27	1.44	1.87	*	1.53
6^b^	0.98	0.97	0.87	0.87	0.92

^a ^responder sample in microarray analysis

^b ^non-responder sample in microarray analysis

*: RNA used in the microarray hybridizations was not available for this test.

### Progesterone receptor (PR) status in the responder and non-responder cases

We explored whether genetic differences in the PR could explain the differential response to P4 in the responder and non-responder OSE cell cultures. It has been suggested that certain genetic polymorphisms in the PR gene could modify the transcriptional activity of the PR [28,29]. These SNPs were V660L, which tags a distinct haplotype known as PROGINS, and +331 G/A, a SNP located in the promoter region. The responder case 2 was heterozygous for both SNPs, whereas the other five cases were homozygous G/G-V/V. Thus, the genotypes of the two SNPs were not significantly different between the responder and non-responder cases. To investigate the expression status of PR, we conducted real-time qRT-PCR analysis using PR primers amplifying the transcript portion common to both the PR-A and PR-B isoforms and specific to the PR-B isoform in the P4-exposed and baseline control cultures. This analysis showed that both isoforms of PR were expressed in the responder and non-responders cell cultures without significant changes upon P4 exposure (Additional File 1).

### Tissue-specific expression levels of *TMEM97 *correlate with cholesterol biosynthesis genes

To determine whether co-expression of *TMEM97 *and cholesterol biosynthesis genes also occurs in other normal tissues, we analyzed tissue-specific expression levels in the GNF Gene Expression Atlas2 database [30] using UCSC Genome Browser's Gene Sorter utility [31]. The Gene Sorter ranks 20,161 human genes based on their difference in tissue-specific expression levels relative to a selected gene, which was ranked at number 1, using data from multiple tissues including fetal brain, whole brain, amygdala, thymus, bone marrow, CD4+ peripheral blood cells, adipocyte, pancreatic isles, heart, lung, kidney, liver, ovary and testes. When *TMEM97 *was ranked at number 1, farnesyl diphosphate synthase (*FDPS*), farnesyl-diphosphate farnesyltransferase (*FDFT1*) and acetyl-CoA acetyltransferase 2 (*ACAT2*) genes were ranked, at numbers 10, 34 and 43, respectively, among the top 50 genes that have the closest tissue-specific expression levels (last column in Additional File 2). The ranking of 3 of the 23 cholesterol biosynthesis genes among the top 50 genes is highly unexpected (expected number = 0.06, observed number = 3; Poisson p-value = 0.00003). Alternatively, the ranking of 18 cholesterol biosynthesis genes among the top 5000 is also highly unexpected (expected number = 6, observed number = 18; Fisher's exact test = 0.00047). These results support our conclusions derived from the microarray analyses of OSE cells in that the expression of *TMEM97 *is co-regulated with cholesterol biosynthesis genes in normal tissues.

### Evaluation of *TMEM97 *in ovarian cancer

Because somatic alterations in downstream targets of P4 in OSE cells could play a role in the pathogenesis of OvCa, we compared the expression of *TMEM97 *in normal OSE cells and OvCa cells that are maintained in the short-term culture and harvested for RNA extraction under identical conditions. We evaluated the expression of *TMEM97 *in OvCa samples and in normal OSE cells relative to the control genes using real-time quantitative RT-PCR. Compared to the normal OSE cells, the OvCa samples showed 2.71- and 2.22-fold reduced expression of *TMEM97 *when SNRP70 and GAPDH were used as control genes, respectively (Table 4).

**Table 4:** qRT-PCR analysis of *TMEM97 *expression in ovarian cancer (OvCa) relative to normal ovarian surface epithelial (OSE) cells

Control gene	ΔC_T _in OSE (n, SE)	ΔC_T _in OvCa (n, SE)	ΔΔC_T _(OvCa-OSE)	*Fold-change of *TMEM97 *(suppression in OvCa)
GAPDH	7.86 (5, 0.48)	9 (28, 0.29)	1.14	2.204
SNRP70	-1.30 (8, 0.90)	0.14 (21, 0.52)	1.44	2.713

ΔC_T _= Average cycle threshold difference between *TMEM97 *and the control gene

n = number of samples

SE = standard error of the mean

*Fold-change in expression = 2^ΔΔCT^

To test for somatic mutations in the *TMEM97 *gene, which is located on chromosome band 17q11.2, we sequenced all of the coding nucleotides (528 base pairs), that are distributed to three exons, in 39 ovarian cancer samples. We found an intronic single nucleotide polymorphism (IVS1 +12 C>T) in a single sample but no coding sequence variations.

## Discussion

To determine the intracellular gene targets of P4 in non-neoplastic OSE cells, we analyzed changes in gene expression patterns in six independent short-term cultures using genome-wide transcript arrays. We found a highly statistically significant regulation of transcripts involved in cholesterol metabolism. For example, multiple transcripts representing cholesterol-homeostasis genes including *HMGCS1, HMGCR, IDI1, FDPS, FDFT1, NSDHL, EBP, DHCR7, INSIG1, FADS1 *were upregulated by P4 exposure (Additional File 2). Examination of each experimental pair revealed that the transcriptional activity induced by P4 exposure originated only from three of the six experimental pairs. Re-analysis of these three responder pairs uncovered a functionally uncharacterized gene *TMEM97 *as the most-responsive transcript which showed a 1.95-fold increase upon P4 exposure. Examination of genome-scale, tissue-specific gene expression levels in the GNF2 database uncovered a strong correlation between *TMEM97 *and cholesterol biosynthesis genes. This finding and our current microarray analyses suggest that *TMEM97 *plays a role in cholesterol metabolism. We also found that the expression of *TMEM97 *was downregulated ~2.4-fold in OvCa samples. Collectively, these findings suggest that P4 is involved in cholesterol metabolism in OSE cells. To our knowledge, P4-regulation of cholesterol homeostasis genes in OSE cells has not been previously suggested. An earlier work has shown suppression of a subset of the cholesterol biosynthesis pathway genes by PR receptor inhibitors in rat periovulatory granulose cells suggesting that P4 regulation of cholesterol homeostasis could be, at least in part, receptor-mediated [32].

The lack of significant gene expression changes in three of the six experimental pairs suggests that the transcriptional response to P4 may not be universal among OSE cells, at least under the experimental conditions described herein. The reasons for this differential response are currently unclear. Analyses of two genetic polymorphisms and the expression of PR gene did not reveal appreciable differences between the responder and non-responder groups. Certain clinical characteristics such as history of oral contraceptive use, serum gonadotropin, estrogen or progesterone levels at the time of surgery might have influenced the responsiveness of the OSE cells to P4. However, because the OSE cells remained in culture under identical conditions for at least two weeks before the experiments, it is unlikely that any reversible *in vivo *effects of these hormones would persist when the cells were treated with P4. Alternatively, certain constitutional or somatically-induced genetic changes might affect the P4-responsiveness of certain OSE cells. Notably, one of the three non-responder OSE cells was derived from a subject (case # 3) whose ovaries were removed because of her sister's history of early-onset ovarian cancer (Table 1). A second non-responder sample (case # 5) was derived from a subject with endometrial cancer, which sometimes co-occurs with OvCa [33]. Thus, whether mutations increasing susceptibility to OvCa could blunt responses to P4 remains to be investigated. Although hormonal and/or genetic factors might affect the responsiveness of certain OSE samples to P4, our data indicate that at least certain OSE samples clearly show a broad transcriptional regulation of cholesterol homeostasis genes upon P4 exposure. Thus, potential impact of cholesterol metabolism on the pathogenesis of OvCa should be considered.

Our data suggest that the impact of P4 on cholesterol metabolism is profound and involves broad transcriptional regulation of many genes regulating cholesterol and lipid biosynthesis and transport. The P4-induced transcriptional changes in most genes in cholesterol metabolism predict an increase in intracellular cholesterol levels. These changes include (a) up-regulation of 14 enzymes catalyzing both early and late steps of *de novo *biosynthesis (b) up-regulation of the low density lipoprotein receptor and ATP-binding cassette (ABC) transporter *ABCG1 *genes [34], which predict increased cellular uptake and (c) reduced conversion to gonadal and adrenal steroids by down-regulation of the *StAR *gene [35]. It is conceivable that increased cholesterol synthesis could compensate for the downregulation of StAR and lead to increased OSE steroid synthesis. Similarly, the P4-induced transcriptional up-regulation of genes involved in unsaturated fatty acid metabolism predicts an increased synthesis of unsaturated fatty acids. Whereas the up-regulation of stearoyl-CoA and fatty acid desaturases predicts an increased *de novo *synthesis [36], up-regulation of the phospholipase A2 gene *PLA2G4A *and endothelial lipase [37] may promote release of unsaturated fatty acids such as oleic acid and arachidonic acid from membrane phospholipids. Similarly, down-regulation of cytochrome P450 gene *CYP2C18 *[38] might help reduce conversion of arachidonic acid to other biologically active eicosanoids.

Cholesterol and fatty acids are essential components of the cell membrane [39]. Thus, the P4-induced changes in cholesterol and lipid metabolism might influence such physicochemical characteristics of the OSE cell membrane as fluidity. OSE cells remain physically intact during pregnancy and the secretory phase of the menstrual cycle, when P4 levels are high and no ovulation occurs. Perhaps, in these physiological states, an increase in the cholesterol content of OSE cell membrane induced by P4 might decrease membrane fluidity and help maintain the physical integrity of the OSE cells that cover the ovary. Notably, inhibitory effects of P4 on membrane fluidity of SKOV-3 ovarian adenocarcinoma cells has been independently suggested and linked to decreased tumorigenic behavior of P4-treated OvCa cells [11]. The present results suggest that increased cholesterol biosynthesis might underlie the decreased membrane fluidity observed in SKOV-3 cells. The current findings might be further interpreted to suggest that defects in the P4-induced cholesterol biosynthesis pathway might increase vulnerability of OSE cells to inflammatory or physical damage and increase the risk of neoplastic transformation. Accordingly, we found that *TMEM97*, the most upregulated gene by P4 in our dataset, was coordinately expressed with cholesterol biosynthesis genes in other tissues and was suppressed 2.4-fold in the OvCa samples relative to normal OSE cells. Further experiments including evaluation of the expressional status of other cholesterol biosynthesis genes and functional studies of cholesterol metabolism genes by employing such experimental approaches as siRNA inhibition of the candidate genes in OvCa cells might help to understand whether alterations in cholesterol homeostasis plays a general role in pathogenesis of ovarian cancer.

## Conclusion

The present results suggest that *TMEM97 *and cholesterol and lipid homeostasis genes are transcriptional targets of P4 in normal OSE cells. If confirmed, these results could provide new insights on the molecular steps involved in the development of OvCa.

## List of abbreviations

OSE cells: Ovarian Surface Epithelial cells; OvCa: Ovarian cancer; P4: Progesterone; PR: Progesterone receptor; qRT-PCR: Quantitative reverse transcription-polymerase chain reaction

## Competing interests

The author(s) declare that they have no competing interests.

## Authors' contributions

BEB and JAD designed the study. BEB, CBW, GOF, JEWB and LCH performed the experiments. BEB and CBW performed the statistical analyses. BEB and JAD obtained funding. CWB and BEB drafted the manuscript. GOF, JEWB and LCH revised the manuscript for important intellectual contribution. All authors read and approved the final manuscript.

## Pre-publication history

The pre-publication history for this paper can be accessed here:



## Supplementary Material

Additional File 1 Progesterone receptor characteristics of ovarian surface epithelial cells exposed to P4. The data provides progesterone receptor (PR) polymorphisms and PR expressional changes to P4 exposure. Click here for file

Additional File 3 Outlier transcripts from the responder samples Click here for file

Additional File 2 Fold-changes in cholesterol homeostasis-related gene expression in normal OSE cells treated with P4. The data describe average fold changes upon P4 exposure of cholesterol homeostasis genes. The data also describe significant similarity in tissue specific expression patterns between TMEM97 (MAC30) and cholesterol and lipid biosynthesis genes. Click here for file
