# Supplementary material for: Coordinate up-regulation of TMEM97 and cholesterol biosynthesis genes in normal ovarian surface epithelial cells treated with progesterone: implications for pathogenesis of ovarian cancer
Source: BMC Cancer. 2007 Dec 11;7:223. doi: 10.1186/1471-2407-7-223 (PMC2241839; doi:10.1186/1471-2407-7-223)
Supplement: Additional File 1 — Progesterone receptor characteristics of ovarian surface epithelial cells exposed to P4. The data provides progesterone receptor (PR) polymorphisms and PR expressional changes to P4 exposure. [file 1471-2407-7-223-S1.doc]

Additional Table 1

Progesterone receptor characteristics of ovarian surface epithelial cells exposed to P4

| Case | Transcriptional response to progesterone | PR +331 (promoter) g/a SNP genotype | PR V660L (exon 4)  SNP genotype | Fold-change in PR (PRA+PRB) expression upon P4 exposure | Fold-change in PRB isoform expression upon P4 exposure |
| --- | --- | --- | --- | --- | --- |
| 1 | Yes | G/G | V/V | Not tested | Not tested |
| 2 | Yes | G/A | V/L | 0.97 | 1.85 |
| 3 | No | G/G | V/V | 1.12 | 1.11 |
| 4 | Yes | G/G | V/V | Not tested | Not tested |
| 5 | No | G/G | V/V | Not tested | Not tested |
| 6 | No | G/G | V/V | 0.66 | 1.28 |
