# Supplementary material for: Coordinate up-regulation of TMEM97 and cholesterol biosynthesis genes in normal ovarian surface epithelial cells treated with progesterone: implications for pathogenesis of ovarian cancer
Source: BMC Cancer. 2007 Dec 11;7:223. doi: 10.1186/1471-2407-7-223 (PMC2241839; doi:10.1186/1471-2407-7-223)
Supplement: Additional File 3 — Outlier transcripts from the responder samples [file 1471-2407-7-223-S3.doc]

**Additional file 3**

**Outlier transcripts from the responder samples**

|  | **Up-regulated outlier transcripts** |  |  |  |
| --- | --- | --- | --- | --- |
| **Probe set** | Gene | **Accession No.** | **Fold change** | **Average raw signal** |
| 212281_s_at | hypothetical protein MAC30 (TMEM97) | L19183.1 | 1.949 | 758.63 |
| 204162_at | kinetochore associated 2 (highly expressed in cancer, rich in leucine heptad repeats) | NM_006101.1 | 1.828 | 125.05 |
| 212282_at | hypothetical protein MAC30 (TMEM97) | L19183.1 | 1.793 | 982.50 |
| 201625_s_at | insulin induced gene 1 | BE300521 | 1.761 | 830.35 |
| 218542_at | hypothetical protein FLJ10540 (C10orf3) | NM_018131.1 | 1.713 | 451.43 |
| 208963_x_at | fatty acid desaturase 1 | BE540552 | 1.712 | 726.10 |
| 219148_at | T-LAK cell-originated protein kinase (TOPK) (PDZbinding kinase) | NM_018492.1 | 1.710 | 251.21 |
| 201627_s_at | insulin induced gene 1 | NM_005542.1 | 1.707 | 1730.78 |
| 218039_at | nucleolar protein ANKT | NM_016359.1 | 1.656 | 564.95 |
| 201890_at | ribonucleotide reductase M2 polypeptide | NM_001034.1 | 1.630 | 473.72 |
| 200832_s_at | stearoyl-CoA desaturase (delta-9-desaturase) | AB032261.1 | 1.624 | 3729.25 |
| 202540_s_at | 3-hydroxy-3-methylglutaryl-Coenzyme A reductase | NM_000859.1 | 1.619 | 707.33 |
| 209608_s_at | acetyl-Coenzyme A acetyltransferase 2 (acetoacetyl Coenzyme A thiolase) | BC000408.1 | 1.609 | 1144.36 |
| 201626_at | insulin induced gene 1 | BE300521 | 1.607 | 1859.47 |
| 218662_s_at | chromosome condensation protein G (HCAP-G) | NM_022346.1 | 1.607 | 124.19 |
| 205027_s_at | mitogen-activated protein kinase kinase kinase 8 | NM_005204.1 | 1.592 | 212.47 |
| 212279_at | hypothetical protein MAC30 | L19183.1 | 1.583 | 967.75 |
| 221750_at | 3-hydroxy-3-methylglutaryl-Coenzyme A synthase 1 (soluble) | BG035985 | 1.577 | 1383.67 |
| 203764_at | Drosophila discs large-1 tumor supressor-like | NM_014750.1 | 1.571 | 245.31 |
| 204256_at | long-chain fatty-acyl elongase | NM_024090.1 | 1.570 | 488.65 |
| 218501_at | Rho guanine nucleotide exchange factor (GEF) 3 | NM_019555.1 | 1.560 | 1095.58 |
| 218755_at | RAB6 interacting, kinesin-like (KIF20A) | NM_005733.1 | 1.551 | 451.45 |
| 219294_at | hypothetical protein FLJ10545 6p12.3 | NM_018132.1 | 1.548 | 67.61 |
| 208881_x_at | isopentenyl-diphosphate delta isomerase | BC005247.1 | 1.544 | 2880.68 |
| 204615_x_at | isopentenyl-diphosphate delta isomerase | NM_004508.1 | 1.519 | 2842.91 |
| 203887_s_at | thrombomodulin (THBD) 20p11.21 | NM_000361.1 | 1.519 | 590.34 |
| 209773_s_at | ribonucleotide reductase M2 polypeptide | BC001886.1 | 1.514 | 681.74 |
| 219181_at | lipase, endothelial | NM_006033.1 | 1.513 | 275.41 |
| 215093_at | NAD(P) dependent steroid dehydrogenase-like; H105e3 | U82671 | 1.511 | 587.63 |
| 207165_at | hyaluronan-mediated motility receptor (RHAMM) | NM_012485.1 | 1.501 | 260.93 |
| 202503_s_at | KIAA0101 gene product 15q22.31 | NM_014736.1 | 1.485 | 862.73 |
| 212276_at | lipin 1 | D80010.1 | 1.475 | 1300.20 |
| 201292_at | topoisomerase (DNA) II alpha 170kDa | NM_001067.1 | 1.473 | 403.84 |
| 48031_r_at | chromosome 5 open reading frame 4 5q33.2 | H93077 | 1.472 | 140.54 |
| 203213_at | cell division cycle 2, G1 to S and G2 to M | AL524035 | 1.468 | 391.53 |
| 201275_at | farnesyl diphosphate synthase (farnesyl pyrophosphate synthetase, dimethylallyltranstransferase, geranyltranstransferase) | NM_002004.1 | 1.462 | 3086.94 |
| 220717_at | ADAMTS20 | NM_025003.1 | 1.462 | 52.02 |
| 202539_s_at | 3-hydroxy-3-methylglutaryl-Coenzyme A reductase | AL518627 | 1.453 | 1044.07 |
| 201791_s_at | 7-dehydrocholesterol reductase | NM_001360.1 | 1.448 | 1470.58 |
| 208964_s_at | fatty acid desaturase 1 | AL512760.1 | 1.447 | 1763.96 |
| 218883_s_at | MLF1 interacting protein 4q35.1 | NM_024629.1 | 1.440 | 354.17 |
| 222149_x_at | Homo sapiens mRNA; cDNA DKFZp434K052 (from clone DKFZp434K052) | AL137398.1 | 1.437 | 105.42 |
| 204567_s_at | ATP-binding cassette, sub-family G (WHITE), member 1….cholesterol efflux to LDL | NM_004915.2 | 1.433 | 143.17 |
| 201790_s_at | 7-dehydrocholesterol reductase | AW150953 | 1.432 | 2109.92 |
| 212274_at | lipin 1 | D80010.1 | 1.428 | 961.10 |
| 204785_x_at | interferon (alpha, beta and omega) receptor 2 | NM_000874.1 | 1.427 | 411.51 |
| 205072_s_at | X-ray repair complementing defective repair in Chinese hamster cells 4 (XRCC4) | NM_022406.1 | 1.425 | 95.31 |
| 213624_at | acid sphingomyelinase-like phosphodiesterase | AA873600 | 1.421 | 640.65 |
| 208808_s_at | high-mobility group box 2 (HMGB2) | BC000903.1 | 1.421 | 556.99 |
| 209279_s_at | NAD(P) dependent steroid dehydrogenase-like; H105e3 | BC000245.1 | 1.418 | 600.74 |
| 214927_at | Homo sapiens mRNA full length insert cDNA clone EUROIMAGE 1968422 | AL359052.1 | 1.416 | 422.92 |
| 220703_at | uncharacterized hypothalamus protein HT009 10p15.3 | NM_018470.1 | 1.416 | 171.66 |
| 210950_s_at | farnesyl-diphosphate farnesyltransferase 1 | BC003573.1 | 1.406 | 3260.70 |
| 204407_at | transcription termination factor, RNA polymerase II | AF080255.1 | 1.405 | 134.91 |
| 208647_at | farnesyl-diphosphate farnesyltransferase 1 | AA872727 | 1.405 | 3755.61 |
| 204727_at | AND-1 protein | AW772140 | 1.400 | 57.20 |
| 219918_s_at | asp (abnormal spindle)-like, microcephaly associated (Drosophila) | NM_018123.1 | 1.398 | 314.85 |
| 210732_s_at | lectin, galactoside-binding, soluble, 8 (galectin 8) 1q43 | AF342816.1 | 1.395 | 275.94 |
| 205440_s_at | neuropeptide Y receptor Y1 4q31.3 | NM_000909.1 | 1.393 | 489.28 |
| 222281_s_at | ESTs, Moderately similar to cytokine receptor-like factor 2; cytokine receptor CRL2 precusor [Homo sapiens] [H.sapiens] | AW517716 | 1.391 | 180.87 |
| 207761_s_at | DKFZP586A0522 protein | NM_014033.1 | 1.390 | 515.40 |
| 202218_s_at | fatty acid desaturase 2 | NM_004265.1 | 1.386 | 1211.07 |
| 203214_x_at | cell division cycle 2, G1 to S and G2 to M | NM_001786.1 | 1.379 | 485.67 |
| 221986_s_at | hypothetical protein FLJ20059 (FROM PROSTATE) | AW006750 | 1.378 | 193.74 |
| 213787_s_at | emopamil binding protein (sterol isomerase) | AV702405 | 1.378 | 1299.78 |
| 202735_at | emopamil binding protein (sterol isomerase) | NM_006579.1 | 1.377 | 746.96 |
| 202314_at | cytochrome P450, 51 (lanosterol 14-alpha-demethylase) | NM_000786.1 | 1.376 | 1090.77 |
| 219564_at | potassium inwardly-rectifying channel, subfamily J, member 16 (KCNJ16) 17q24.3 | NM_018658.1 | 1.366 | 365.80 |
| 219836_at | ZBED2 (zinc finger, BED domain containing 2) | NM_024508.1 | 1.364 | 836.96 |
| 207361_at | HMG-box containing protein 1 (HBP1) | NM_012257.1 | 1.363 | 102.09 |
| 209980_s_at | serine hydroxymethyltransferase 1 (soluble) | L23928.1 | 1.361 | 840.44 |
| 215008_at | tolloid-like 2 | AA582404 | 1.360 | 125.89 |
| 209211_at | Kruppel-like factor 5 (intestinal) (KLF5) | AF132818.1 | 1.360 | 181.58 |
| 213094_at | hypothetical protein DKFZp564D0462 | AL033377 | 1.360 | 323.50 |
| 52285_f_at | hypothetical protein FLJ12542 | AW002970 | 1.360 | 177.09 |
| 209989_at | zinc finger protein 268 | AF317549.1 | 1.357 | 226.62 |
| 216393_at | GPR126 (G protein-coupled receptor 126) 6q24.2 | AL049938.1 | 1.354 | 240.00 |
| 202067_s_at | low density lipoprotein receptor (familial hypercholesterolemia) | AI861942 | 1.352 | 299.27 |
| 213424_at | KIAA0895 protein | AB020702.1 | 1.352 | 67.15 |
| 220894_x_at | PR domain containing 12 (PRDM12) | NM_021619.1 | 1.351 | 228.58 |
| 209183_s_at | C10orf10 (chromosome 10 open reading frame 10) | AL136653.1 | 1.348 | 1909.16 |
| 202388_at | regulator of G-protein signalling 2, 24kDa (RGS2) | NM_002923.1 | 1.348 | 499.99 |
| 217989_at | 17-beta-hydroxysteroid dehydrogenase type XI | NM_016245.1 | 1.346 | 1102.30 |
| 207828_s_at | centromere protein F, 350/400ka (mitosin) | NM_005196.1 | 1.345 | 412.18 |
| 206001_at | neuropeptide Y | NM_000905.1 | 1.343 | 298.66 |
| 213915_at | natural killer cell group 7 sequence (NKG7) | NM_005601.1 | 1.342 | 197.83 |
| 205239_at | amphiregulin (schwannoma-derived growth factor) | NM_001657.1 | 1.338 | 237.12 |
| 203407_at | periplakin | NM_002705.1 | 1.337 | 1337.91 |
| 203967_at | CDC6 cell division cycle 6 homolog (S. cerevisiae) | U77949.1 | 1.335 | 197.63 |
| 202705_at | cyclin B2 | NM_004701.2 | 1.334 | 332.89 |
| 217232_x_at | hemoglobin, beta | AF059180 | 1.332 | 1737.19 |
| 201193_at | isocitrate dehydrogenase 1 (NADP+), soluble | NM_005896.1 | 1.331 | 2541.05 |
| 209146_at | sterol-C4-methyl oxidase-like | AV704962 | 1.331 | 3339.54 |
| 218009_s_at | protein regulator of cytokinesis 1 | NM_003981.1 | 1.331 | 509.64 |
| 210145_at | phospholipase A2, group IVA (cytosolic, calcium-dependent) | M68874.1 | 1.330 | 173.46 |
| 202245_at | lanosterol synthase (2,3-oxidosqualene-lanosterol cyclase) | AW084510 | 1.330 | 2599.75 |
| 201291_s_at | topoisomerase (DNA) II alpha 170kDa | NM_001067.1 | 1.330 | 215.62 |
| 218726_at | hypothetical protein DKFZp762E1312 | NM_018410.1 | 1.330 | 250.63 |
| 208961_s_at | core promoter element binding protein (COPEB) | AB017493.1 | 1.329 | 2106.89 |
| 202083_s_at | SEC14-like 1 (S. cerevisiae) | NM_003003.1 | 1.326 | 283.67 |
| 214710_s_at | cyclin B1 | BE407516 | 1.326 | 535.87 |
| 208216_at | distal-less homeobox 4 (DLX4) 17q21.33 | NM_001934.1 | 1.326 | 187.95 |
| 219166_at | hypothetical protein FLJ10563 | NM_018139.1 | 1.324 | 187.08 |
| 206282_at | Homo sapiens cDNA FLJ12248 fis, clone MAMMA1001408 | AU147698 | 1.323 | 80.81 |
| 214155_s_at | c-Mpl binding protein | AI743740 | 1.316 | 253.39 |
| 205943_at | tryptophan 2,3-dioxygenase | NM_005651.1 | 1.313 | 103.58 |
| 221911_at | ETV1 (ets variant gene 1) | AF070641.1 | 1.312 | 518.78 |
| 219493_at | hypothetical protein FLJ22009 | NM_024745.1 | 1.310 | 370.91 |
| 204240_s_at | SMC2 structural maintenance of chromosomes 2-like 1 (yeast) (SMC2L1) | NM_006444.1 | 1.309 | 455.03 |
| 219804_at | SYNPO2L (synaptopodin 2-like) | NM_024875.1 | 1.308 | 438.78 |
| 218355_at | kinesin family member 4A (KIF4A) | NM_012310.2 | 1.308 | 213.40 |
| 212709_at | nucleoporin 160kDa | D83781.1 | 1.307 | 237.27 |
| 204159_at | cyclin-dependent kinase inhibitor 2C (p18, inhibits CDK4) | NM_001262.1 | 1.306 | 259.76 |
| 207195_at | enhancer of zeste homolog 2 (Drosophila) (EZH2) | NM_004456.1 | 1.306 | 74.51 |
| 202870_s_at | CDC20 cell division cycle 20 homolog (S. cerevisiae) | NM_001255.1 | 1.304 | 394.48 |
| 212094_at | paternally expressed 10 (PEG10) | BE858180 | 1.302 | 295.29 |
| 213246_at | DKFZP564F1123 protein | AI346504 | 1.302 | 521.39 |
| 206834_at | hemoglobin, delta | NM_000519.2 | 1.298 | 3147.72 |
| 203665_at | heme oxygenase (decycling) 1 | NM_002133.1 | 1.298 | 1989.45 |
| 213007_at | hypothetical protein FLJ10719 | BG478677 | 1.297 | 221.58 |
| 202589_at | thymidylate synthetase | NM_001071.1 | 1.297 | 1379.24 |
| 214700_x_at | DKFZP434D193 protein | AK000323.1 | 1.295 | 316.94 |
| 218663_at | chromosome condensation protein G (HCAP-G) | NM_022346.1 | 1.293 | 111.58 |
| 209116_x_at | hemoglobin, beta | M25079.1 | 1.293 | 1537.42 |
| 216411_s_at | galactokinase 2 | AK023699.1 | 1.293 | 301.06 |
| 219174_at | CCDC2 (coiled-coil domain containing 2) | NM_025103.1 | 1.292 | 169.25 |
| 219164_s_at | hypothetical protein FLJ10242 (C14orf103) | NM_018036.1 | 1.291 | 330.59 |
| 218929_at | hypothetical protein FLJ20036 | NM_017632.1 | 1.291 | 366.53 |
| 202338_at | thymidine kinase 1, soluble | NM_003258.1 | 1.291 | 311.03 |
| 211423_s_at | sterol-C5-desaturase (ERG3 delta-5-desaturase homolog, fungal)-like | D85181.1 | 1.289 | 2415.32 |
| 201739_at | serum/glucocorticoid regulated kinase | NM_005627.1 | 1.289 | 2110.46 |
| 220038_at | serum/glucocorticoid regulated kinase-like | NM_013257.1 | 1.288 | 219.26 |
| 53202_at | hypothetical protein MGC2821 | AA402435 | 1.288 | 87.08 |
| 213679_at | DKFZP564G196 protein | AL049329.1 | 1.286 | 119.74 |
| 219691_at | hypothetical protein FLJ20073 | NM_017654.1 | 1.285 | 303.65 |
| 61732_r_at | coiled-coil domain containing 2 | AI610355 | 1.285 | 137.62 |
| 207387_s_at | glycerol kinase | NM_000167.1 | 1.285 | 109.87 |
| 205835_s_at | hypothetical protein FLJ21940 | AW975818 | 1.284 | 122.24 |
| 205822_s_at | 3-hydroxy-3-methylglutaryl-Coenzyme A synthase 1 (soluble) | NM_002130.1 | 1.284 | 306.87 |
| 219487_at | hypothetical protein FLJ23560 | NM_024685.1 | 1.280 | 117.88 |
| 220817_at | transient receptor potential cation channel, subfamily C, member 4 (TRPC4) | NM_016179.1 | 1.280 | 144.55 |
| 201633_s_at | cytochrome b5 outer mitochondrial membrane precursor | AW235051 | 1.279 | 296.62 |
| 206005_s_at | KIAA1009 protein | AK023613.1 | 1.279 | 143.21 |
| 202843_at | DnaJ (Hsp40) homolog, subfamily B, member 9 (DNAJB9) | NM_012328.1 | 1.279 | 387.53 |
| 222268_x_at | (MUC5B) mucin 5, subtype B, tracheobronchial | AA587390 | 1.279 | 333.65 |
| 206300_s_at | parathyroid hormone-like hormone | NM_002820.1 | 1.279 | 199.95 |
| 220351_at | chemokine (C-C motif) receptor-like 1 | NM_016557.1 | 1.278 | 88.03 |
| 209377_s_at | high mobility group nucleosomal binding domain 3 (HMGN3) | AF274949.1 | 1.278 | 4413.04 |
| 216497_at | GSH1 | AL390738 | 1.277 | 195.53 |
| 205309_at | acid sphingomyelinase-like phosphodiesterase | NM_014474.1 | 1.276 | 356.80 |
| 204774_at | ecotropic viral integration site 2A (EVI2A) | NM_014210.1 | 1.275 | 304.14 |
| 206026_s_at | tumor necrosis factor, alpha-induced protein 6 (TNFAIP6) | NM_007115.1 | 1.271 | 214.66 |
| 212755_at | KIAA1040 protein | AI760249 | 1.271 | 218.96 |
| 213761_at | nuclear protein double minute 1 (Mdm1) | AW664850 | 1.271 | 237.79 |
| 217546_at | MT1K = metallothionein 1K | R06655 | 1.271 | 216.10 |
| 221193_s_at | hypothetical protein FLJ20094 | NM_017665.1 | 1.271 | 294.77 |
| 217672_x_at | ESTs | BF114906 | 1.269 | 286.00 |
| 219555_s_at | uncharacterized bone marrow protein BM039 | NM_018455.1 | 1.269 | 202.86 |
| 203493_s_at | translokin | AI123527 | 1.269 | 222.84 |
| 214974_x_at | chemokine (C-X-C motif) ligand 5 (CXCL5) | AK026546.1 | 1.267 | 426.50 |
| 219694_at | hypothetical protein FLJ11127 | NM_019018.1 | 1.265 | 191.16 |
| 205836_s_at | YTH domain containing 2 (YTHDC2) | NM_022828.1 | 1.264 | 230.95 |
| 215046_at | hypothetical protein FLJ23861 | AL133053.1 | 1.264 | 227.83 |
| 220480_at | heart and neural crest derivatives expressed 2 (HAND2) | NM_021973.1 | 1.263 | 198.97 |
| 208762_at | ubiquitin-like 1 (sentrin) (UBL1) | U83117.1 | 1.262 | 171.26 |
| 213984_at | KIAA0648 protein | AW991219 | 1.262 | 99.46 |
| 219688_at | BBS7 (Bardet-Biedl syndrome 7) | NM_018190.1 | 1.258 | 171.59 |
| 217344_at | Consensus includes gb:AL022163 /DEF=Human DNA sequence from clone 551E13 on chromosome Xp11.2-11.3 Contains farnesyl pyrophosphate synthetase pseudogene, VT4 protein pseudogene, EST, GSS | AL022163 | 1.258 | 353.75 |
| 222359_x_at | (FVT1) 3-ketodihydrosphingosine reductase | BF573849 | 1.253 | 108.81 |
| 209314_s_at | HBS1-like (S. cerevisiae) | AK024258.1 | 1.250 | 162.12 |
| 206451_at | hypothetical protein FLJ10560 | NM_018138.1 | 1.247 | 204.46 |
| 215649_s_at | mevalonate kinase (mevalonic aciduria) | AF217536.1 | 1.247 | 153.94 |
| 202890_at | microtubule-associated protein 7 (MAP7) | T62571 | 1.246 | 209.69 |
| 203347_s_at | likely ortholog of mouse metal response element binding transcription factor 2 (M96) | NM_007358.1 | 1.245 | 122.48 |
| 204291_at | KIAA0335 gene product | NM_014803.1 | 1.245 | 159.09 |
| 209172_s_at | centromere protein F, 350/400ka (mitosin) | U30872.1 | 1.244 | 181.44 |
| 203352_at | origin recognition complex, subunit 4-like (yeast) | NM_002552.1 | 1.237 | 206.91 |
| 210553_x_at | paired basic amino acid cleaving system 4 (PACE4) | D28514.1 | 1.226 | 164.77 |
|  | **Down-regulated outlier transcripts** |  |  |  |
| **Probe set** | **Gene** | **Accession No.** | **Fold change** | **Average raw signal** |
| 211959_at | Human insulin-like growth factor binding protein 5 (IGFBP5) mRNA | L27560.1 | 0.481 | 506.97 |
| 214701_s_at | fibronectin 1 | AJ276395.1 | 0.590 | 4634.95 |
| 215513_at | hydatidiform mole associated and imprinted | AF241534.1 | 0.607 | 152.38 |
| 211876_x_at | protocadherin gamma subfamily A, 3,5,6,10,11,12 | AF152504.1 | 0.636 | 371.82 |
| 216352_x_at | protocadherin gamma subfamily A, 3 | AF152509.1 | 0.644 | 341.88 |
| 220154_at | bullous pemphigoid antigen 1, 230/240kDa (BPAG1) | NM_020388.1 | 0.648 | 93.68 |
| 221900_at | collagen, type VIII, alpha 2 (COL8A2) | AI806793 | 0.667 | 245.07 |
| 204585_s_at | L1 cell adhesion molecule (hydrocephalus, stenosis of aqueduct of Sylvius 1, MASA (mental retardation, aphasia, shuffling gait and adducted thumbs) syndrome, spastic paraplegia 1) | NM_000425.2 | 0.670 | 528.89 |
| 215559_at | ATP-binding cassette, sub-family C (CFTR/MRP), member 6 (ABCC6) | AI074459 | 0.673 | 235.15 |
| 205410_s_at | ATPase, Ca++ transporting, plasma membrane 4 | NM_001684.1 | 0.691 | 285.26 |
| 212143_s_at | insulin-like growth factor binding protein 3 | NM_000598.1 | 0.692 | 865.82 |
| 202718_at | insulin-like growth factor binding protein 2, 36kDa | NM_000597.1 | 0.694 | 1087.93 |
| 211668_s_at | plasminogen activator, urokinase (PLAU) | K03226.1 | 0.700 | 1153.02 |
| 212937_s_at | collagen, type VI, alpha 1 | BE350145 | 0.700 | 275.00 |
| 204130_at | hydroxysteroid (11-beta) dehydrogenase 2 | NM_000196.1 | 0.704 | 183.79 |
| 206660_at | immunoglobulin lambda-like polypeptide 1 | NM_020070.1 | 0.704 | 250.92 |
| AFFX-r2-Hs28SrRNA-3_at | M11167 Human 28S rRNA sequence, length 5025 bases, middle target bases 1666-3330 | M11167 | 0.708 | 1019.58 |
| 206275_s_at | flavoprotein oxidoreductase MICAL2 | NM_014632.1 | 0.708 | 407.91 |
| 202222_s_at | desmin | NM_001927.1 | 0.709 | 769.33 |
| 205952_at | potassium channel, subfamily K, member 3 (KCNK3) | NM_002246.1 | 0.712 | 267.17 |
| 217699_at | ESTs, Weakly similar to cytokine receptor-like factor 2; cytokine receptor CRL2 precusor [Homo sapiens] [H.sapiens] | AV700338 | 0.713 | 63.65 |
| 212091_s_at | collagen, type VI, alpha 1 | X99135 | 0.715 | 728.30 |
| 207274_at | cholinergic receptor, nicotinic, epsilon polypeptide | NM_000080.1 | 0.716 | 271.05 |
| 204455_at | dystonin | NM_001723.1 | 0.720 | 62.81 |
| 203627_at | insulin-like growth factor 1 receptor | NM_000875.2 | 0.721 | 1001.50 |
| 206825_at | oxytocin receptor | NM_000916.2 | 0.726 | 3569.87 |
| 210095_s_at | insulin-like growth factor binding protein 3 | M31159.1 | 0.729 | 1668.61 |
| 204570_at | cytochrome c oxidase subunit VIIa polypeptide 1 (muscle) (COX7A1) | NM_001864.1 | 0.732 | 322.46 |
| 202311_s_at | collagen, type I, alpha 1 | NM_000088.1 | 0.733 | 1647.93 |
| 212081_x_at | HLA-B associated transcript 2 (BAT2) | AF129756 | 0.735 | 797.20 |
| 216112_at | ZNF532 (zinc finger protein 532) | AU157200 | 0.737 | 296.72 |
| 217557_s_at | carboxypeptidase M | NM_001874.1 | 0.737 | 98.10 |
| 205479_s_at | plasminogen activator, urokinase | NM_002658.1 | 0.739 | 1897.00 |
| 206382_s_at | brain-derived neurotrophic factor (BDNF) | NM_001709.1 | 0.740 | 744.32 |
| 211880_x_at | protocadherin gamma subfamily A, 1 (PCDHGA1) | AF152507.1 | 0.740 | 251.13 |
| 33767_at | neurofilament, heavy polypeptide 200kDa (NEFH) | X15306 | 0.741 | 165.74 |
| 220594_at | (OGT) O-linked N-acetylglucosamine (GlcNAc) transferase | NM_025192.1 | 0.741 | 131.62 |
| 203434_s_at | membrane metallo-endopeptidase (neutral endopeptidase, enkephalinase, CALLA, CD10) | AI433463 | 0.743 | 1083.09 |
| 217369_at | Similar to Immuglobulin M chain LOC440361 |  | 0.743 | 1157.28 |
| 214722_at | NOTCH2 (Notch homolog 2 (Drosophila)) | AW516297 | 0.744 | 1356.21 |
| 202997_s_at | lysyl oxidase-like 2 (LOXL2) | BE251211 | 0.744 | 473.55 |
| 208982_at | platelet/endothelial cell adhesion molecule (CD31 antigen) (PECAM1) | AW574504 | 0.746 | 393.79 |
| 213606_s_at | Rho GDP dissociation inhibitor (GDI) alpha | AI571798 | 0.748 | 201.28 |
| 214826_at | hypothetical protein DKFZp667B1218 | U79276.1 | 0.749 | 384.17 |
| 217430_x_at | collagen, type I, alpha 1 | Y15916.1 | 0.750 | 2571.68 |
| 220293_at | hypothetical protein FLJ14298 | NM_024764.1 | 0.751 | 118.55 |
| 202015_x_at | methionyl aminopeptidase 2 | NM_006838.1 | 0.752 | 99.23 |
| 205951_at | myosin, heavy polypeptide 1, skeletal muscle, adult | NM_005963.2 | 0.756 | 153.16 |
| 204345_at | collagen, type XVI, alpha 1 | NM_001856.1 | 0.756 | 413.03 |
| AFFX-r2-Hs18SrRNA-M_x_at | M10098 Human 18S rRNA sequence, length 1969 bases, middle target bases 647-1292 | M10098 | 0.756 | 320.92 |
| 202291_s_at | matrix Gla protein | NM_000900.1 | 0.756 | 323.88 |
| 214266_s_at | enigma (LIM domain protein) | AW206786 | 0.756 | 397.29 |
| 213593_s_at | transformer-2 alpha (htra-2 alpha) (TRA2A) | AW978896 | 0.756 | 135.08 |
| 208476_s_at | FRMD4 (FERM domain containing 4) | NM_018027.1 | 0.757 | 117.82 |
| 221730_at | collagen, type V, alpha 2 (COL5A2) | NM_000393.1 | 0.758 | 2555.22 |
| 202998_s_at | lysyl oxidase-like 2 (LOXL2) | NM_002318.1 | 0.759 | 2320.48 |
| 204971_at | cystatin A (stefin A) | NM_005213.1 | 0.760 | 160.33 |
| AFFX-HUMRGE/M10098_3_at | M10098 Human 18S rRNA gene, complete (_5, _M, _3 represent transcript regions 5 prime, Middle, and 3 prime respectively) | M10098 | 0.761 | 1963.37 |
| AFFX-M27830_5_at | M27830 Human 28S ribosomal RNA gene, complete cds (_5, _M, _3 represent transcript regions 5 prime, Middle, and 3 prime respectively) | M27830 | 0.761 | 585.29 |
| 207870_at | A kinase (PRKA) anchor protein (yotiao) 9 (AKAP9) | NM_005751.1 | 0.761 | 140.04 |
| 204320_at | collagen, type XI, alpha 1 | NM_001854.1 | 0.764 | 607.62 |
| 210511_s_at | inhibin, beta A (activin A, activin AB alpha polypeptide) | M13436.1 | 0.765 | 1227.65 |
| 208126_s_at | cytochrome P450, subfamily IIC (mephenytoin 4-hydroxylase), polypeptide 18 (CYP2C18) | NM_000772.1 | 0.765 | 111.10 |
| 203370_s_at | enigma (LIM domain protein) | NM_005451.2 | 0.765 | 1239.55 |
| 214218_s_at | Homo sapiens cDNA FLJ30298 fis, clone BRACE2003172 | AV699347 | 0.767 | 775.26 |
| 213920_at | CUTL2 (cut-like 2 (Drosophila)) | AB006631.1 | 0.767 | 181.71 |
| AFFX-HUMRGE/M10098_5_at | M10098 Human 18S rRNA gene, complete (_5, _M, _3 represent transcript regions 5 prime, Middle, and 3 prime respectively) | M10098 | 0.768 | 383.48 |
| 203369_x_at | enigma (LIM domain protein) | NM_005451.2 | 0.769 | 363.15 |
| 221089_at | NADPH oxidase 3 (NOX3) | NM_015718.1 | 0.769 | 124.86 |
| 221881_s_at | chloride intracellular channel 4 | AI638420 | 0.771 | 2667.28 |
| 201110_s_at | thrombospondin 1 | NM_003246.1 | 0.772 | 684.14 |
| 219028_at | homeodomain interacting protein kinase 2 (HIPK2) | NM_022740.1 | 0.773 | 188.43 |
| 218638_s_at | spondin 2, extracellular matrix protein | NM_012445.1 | 0.778 | 2030.11 |
| 222020_s_at | neurotrimin | AW117456 | 0.779 | 317.67 |
| 204548_at | steroidogenic acute regulatory protein | NM_000349.1 | 0.779 | 947.74 |
| 201069_at | matrix metalloproteinase 2 (gelatinase A, 72kDa gelatinase, 72kDa type IV collagenase) | NM_004530.1 | 0.779 | 3891.47 |
| 202687_s_at | tumor necrosis factor (ligand) superfamily, member 10 (TNFSF10) | U57059.1 | 0.781 | 437.14 |
| 201373_at | plectin 1, intermediate filament binding protein 500kDa | NM_000445.1 | 0.784 | 2076.47 |
| 202771_at | KIAA0233 gene product | NM_014745.1 | 0.790 | 2201.98 |
| 201058_s_at | myosin, light polypeptide 9, regulatory | NM_006097.1 | 0.791 | 4694.45 |
| 202310_s_at | collagen, type I, alpha 1 | NM_000088.1 | 0.796 | 7353.50 |
| 208712_at | cyclin D1 (PRAD1: parathyroid adenomatosis 1) | M73554.1 | 0.802 | 1542.18 |
| 202404_s_at | collagen, type I, alpha 2 | NM_000089.1 | 0.815 | 6730.45 |
| 202403_s_at | collagen, type I, alpha 2 | AA788711 | 0.817 | 10105.82 |

|  |  |  |  |  |  |  |
| --- | --- | --- | --- | --- | --- | --- |
|  |  |  |  |  |  |  |
|  |  |  |  |  |  |  |
|  |  |  |  |  |  |  |
|  |  |  |  |  |  |  |
|  |  |  |  |  |  |  |
|  |  |  |  |  |  |  |
|  |  |  |  |  |  |  |
|  |  |  |  |  |  |  |
|  |  |  |  |  |  |  |
|  |  |  |  |  |  |  |
|  |  |  |  |  |  |  |
|  |  |  |  |  |  |  |
|  |  |  |  |  |  |  |
|  |  |  |  |  |  |  |
|  |  |  |  |  |  |  |
|  |  |  |  |  |  |  |
|  |  |  |  |  |  |  |
|  |  |  |  |  |  |  |
|  |  |  |  |  |  |  |
|  |  |  |  |  |  |  |
|  |  |  |  |  |  |  |
|  |  |  |  |  |  |  |
|  |  |  |  |  |  |  |
|  |  |  |  |  |  |  |
|  |  |  |  |  |  |  |
|  |  |  |  |  |  |  |
|  |  |  |  |  |  |  |
|  |  |  |  |  |  |  |
|  |  |  |  |  |  |  |
|  |  |  |  |  |  |  |
|  |  |  |  |  |  |  |
|  |  |  |  |  |  |  |
|  |  |  |  |  |  |  |
|  |  |  |  |  |  |  |
|  |  |  |  |  |  |  |
|  |  |  |  |  |  |  |
|  |  |  |  |  |  |  |
|  |  |  |  |  |  |  |
|  |  |  |  |  |  |  |
|  |  |  |  |  |  |  |
|  |  |  |  |  |  |  |
|  |  |  |  |  |  |  |
|  |  |  |  |  |  |  |
|  |  |  |  |  |  |  |
|  |  |  |  |  |  |  |
