# Supplementary material for: Coordinate up-regulation of TMEM97 and cholesterol biosynthesis genes in normal ovarian surface epithelial cells treated with progesterone: implications for pathogenesis of ovarian cancer
Source: BMC Cancer. 2007 Dec 11;7:223. doi: 10.1186/1471-2407-7-223 (PMC2241839; doi:10.1186/1471-2407-7-223)
Supplement: Additional File 2 — Fold-changes in cholesterol homeostasis-related gene expression in normal OSE cells treated with P4. The data describe average fold changes upon P4 exposure of cholesterol homeostasis genes. The data also describe significant similarity in tissue specific expression patterns between TMEM97 (MAC30) and cholesterol and lipid biosynthesis genes. [file 1471-2407-7-223-S2.doc]

# Additional File 2

# Fold-changes in cholesterol homeostasis-related gene expression in normal OSE cells treated with P4

| **Cholesterol Homeostasis Gene**^a^ | **Gene Symbol** | **Accession Number** | **Function in cholesterol homeostasis [reference]** | **Average fold change in responders^b^** | **Average fold**  **change in non-responders^b^** | **Similarity to *TMEM97* in Tissue-Specific Expression among 20,161 transcripts in gnf2 database** |
| --- | --- | --- | --- | --- | --- | --- |
| Acetyl-CoA acetyltransferase 2^c^ | ***ACAT2*** | BC000408.1 | Enzyme in Biosynthesis [40] | **1.61^c^** | 0.904 | 43 |
| **HMG CoA Synthase 1 (soluble)^c^** | ***HMGCS1*** | BG035985  NM_002130.1 | Enzyme in Biosynthesis [40] | **1.58^c^**  **1.28^c^** | 0.860  0.996 | 4987 |
| HMG CoA Synthase 2 (mitochondrial) | *HMGCS2* | NM_005518.1 | Enzyme in Biosynthesis [40] | 0.941 | 0.966 | 4987 |
| HMG CoA Reductase^c^ | *HMGCR* | NM_000859.1  AL518627 | Enzyme in Biosynthesis [40] | **1.62^c^**  **1.45^c^** | 0.835  0.880 | 4989 |
| AMP kinase, α1 catalytic subunit | *PRKAA1* | AF100763.1  AK024252.1 | Enzyme in Biosynthesis [40] | 1.090  0.979 | 0.948  0.847 | 630 |
| AMP kinase, α2 catalytic subunit | *PRKAA2* | NM_006252.1 | Enzyme in Biosynthesis [40] | 0.972 | 0.917 | 631 |
| AMP kinase,β1 non-catalyt subunit | *PRKAB1* | BC001007.1  NM_006253.1 | Enzyme in Biosynthesis [40] | 1.001  0.945 | 1.032  0.991 | 624 |
| AMP kinase,β2 non-catalyt subunit | *PRKAB2* | NM_005399.1 | Enzyme in Biosynthesis [40] | 0.911 | 0.939 | 625 |
| AMP kinase, γ1 non-catalyt subunit | *PRKAG1* | NM_002733.1 | Enzyme in Biosynthesis [40] | 0.998 | 1.054 | 626 |
| AMP kinase, γ2 non-catalyt subunit | *PRKAG2* | NM_016203.1 | Enzyme in Biosynthesis [40] | 0.994 | 1.029 | 627 |
| Mevalonate kinase^c^ | ***MVK*** | M88468  NM_000431.1  AF217536.1 | Enzyme in Biosynthesis [40] | 0.960  1.052  **1.25^c^** | 1.084  0.975  1.042 | 5815 |
| Phosphomevalonate kinase | *PMVK* | NM_006556.1 | Enzyme in Biosynthesis [40] | 0.961 | 1.064 | 210 |
| Mevalonate decarboxylase | *MVD* | AI189359 | Enzyme in Biosynthesis [40] | 1.175 | 0.988 | 3741 |
| **Isopentenyl-diphosphate delta isomerase^c^** | ***IDI1*** | NM_004508.1  BC005247.1 | Enzyme in Biosynthesis [40] | **1.519^c^**  **1.544^c^** | 0.983  1.004 | 441 |
| Farnesyl diphosphate synthase^c^ | ***FDPS*** | NM_002004.1  AL022163 | Enzyme in Biosynthesis [40] | **1.462^c^**  **1.258^c^** | 1.022  0.998 | 10 |
| Geranylgeranyl diphosphate synthase 1 | *GGPS1* | AW299507  NM_004837.1 | Enzyme in Biosynthesis [40] | 0.925  0.967 | 0.878  0.952 | 4422 |
| Farnesyl-diphosphate farnesyltransferase^c^ | ***FDFT1*** | AA872727  BC003573.1 | Enzyme in Biosynthesis [40] | **1.40^c^**  **1.41^c^** | 0.965  1.006 | 34 |
| Squalene epoxidase | *SQLE* | AF098865.1  AA639705  AA639705 | Enzyme in Biosynthesis [40] | 1.253  1.284  1.120 | 0.898  0.929  1.064 | NL |
| SEC14-like 1^c^ | ***SEC14L1*** | NM_003003.1  NM_003003.1  NM_003003.1 | Enzyme in Biosynthesis [40] | 0.993  **1.326^c^**  1.132 | 1.109  0.969  1.066 | 17449 |
| Lanosterol synthase^c^ | ***LSS*** | AW084510  D63807.1  D63807.1 | Enzyme in Biosynthesis [40] | **1.330^c^**  1.228  1.086 | 0.977  0.961  0.975 | 3746 |
| Cytochrome P450, 51^c^ **(lanosterol 14-alpha-demethylase)** | ***CYP51A***  *CYP51P2* | NM_000786.1  U40053 | Enzyme in Biosynthesis [40] | **1.376^c^**  1.289 | 0.888  1.041 | 2702 |
| Transmembrane 7 superfamily member 2 (sterol Δ14-reductase) | *TM7SF2* | AF096304.1 | Enzyme in Biosynthesis [40] | 1.019 | 0.879 | 3742 |
| Diaphorase (NADH)  (cytochrome b-5 reductase) | *DIA1* | NM_000398.3 | Enzyme in Biosynthesis [40] | 0.996 | 1.010 | 16615 |
| Sterol-C4-methyl oxidase-like^c^ | ***SC4MOL*** | AV704962 | Enzyme in Biosynthesis [40] | **1.331^c^** | 0.905 | 3743 |
| NAD(P) dependent steroid dehydrogenase-like^c^ | ***NSDHL*** | BC000245.1  U82671 | Enzyme in Biosynthesis [40] | **1.418^c^**  **1.511^c^** | 1.019  0.972 | 7257 |
| Hydroxysteroid (17β) dehydrogenase 7 | *HSD17B7* | NM_016371.1 | Enzyme in Biosynthesis [40] | 1.175 | 1.028 | 3249 |
| **3β-HSD-Δ^h^,Δ^g^-isomerase^c^** | ***EBP*** | NM_006579.1  AV702405  AV702405 | Enzyme in Biosynthesis [40] | **1.377^c^**  **1.378^c^**  1.279 | 1.023  1.033  0.882 | 3534 |
| **3β-HSD-Δ^e^-(sterol C5) desaturase^c^** | ***SC5D*** | D85181.1 | Enzyme in Biosynthesis [40] | **1.289^c^** | 0.976 | 17572 |
| **7-Dehydrocholesterol reductase^c^** | ***DHCR7*** | AW150953  NM_001360.1 | Enzyme in Biosynthesis [40] | **1.432^c^**  **1.448^c^** | 1.026  1.037 | 141 |
| 24-dehydrocholesterol reductase | *DHCR24* | NM_014762.1 | Enzyme in Biosynthesis [40] | 1.059 | 1.054 | 3253 |
| SREBP Cleavage-Activating Protein | *SCAP* | D83782.1 | Enzyme in Biosynthesis [40] | 1.003 | 0.998 | 7406 |
| Insulin induced gene 1^c^ | ***INSIG1*** | BE300521  BE300521  NM_005542.1 | Enzyme in Biosynthesis [40] | **1.761^c^**  **1.607^c^**  **1.707^c^** | 0.978  0.917  0.974 | 79 |
| Sterol regulatory element binding transcription factor 1 | *SREBF1* | NM_004176.1 | Transcriptional activator of cholesterol biosynthesis genes [41] | 1.259 | 1.001 | 18150 |
| Sterol regulatory element binding transcription factor 2 | *SREBF2* | NM_004599.1 | Transcriptional activator of cholesterol and lipid biosynthesis genes [41] | 1.071 | 1.037 | 18151 |
| **ATP-binding cassette, sub-family C6^c^** | ***ABCC6*** | AI074459  AI084637  NM_001171.2 | Cholesterol efflux to HDL [42] | **0.673^c^**  0.892  0.921 | 0.941  0.962  1.092 | 14599 |
| **ATP-binding cassette, sub-family G (WHITE), member 1^c^** | ***ABCG1*** | NM_004915.2  U34919.1 | Cholesterol influx from LDL [34] | **1.433^c^**  0.950 | 1.031  1.057 | 7458 |
| Low density lipoprotein receptor^c^ | ***LDLR*** | AI861942  NM_000527.2 | Cholesterol influx from LDL [40] | **1.352^c^**  1.254 | 0.942  1.014 | 6045 |
| Stearoyl-CoA desaturase ^c^ **(Δ^9^-desaturase)** | ***SCD*** | AB032261.1  AA678241 | Unsaturated fatty acid synthesis [40] | **1.624^c^**  1.230 | 1.061  1.049 | 722 |
| Fatty acid desaturase 1^c^ **(Δ^5^-desaturase)** | ***FADS1*** | BE540552  BE540552  AL512760.1 | Unsaturated fatty acid synthesis [40] | 1.219  **1.712^c^**  **1.447^c^** | 1.001  1.055  0.979 | 13593 |
| Fatty acid desaturase 2^c^ **(Δ^6^-desaturase)** | ***FADS2*** | NM_004265.1 | Unsaturated fatty acid synthesis [40] | **1.386^c^** | 1.127 | 7634 |
| Lipase, endothelial^c^ | ***LIPG*** | NM_006033.1 | Hydrolysis of HDL phospholipids [37] | **1.513^c^** | 0.994 | 6104 |
| **Phospholipase A2, group IVA^c^** | ***PLA2G4A*** | M68874.1 | Hydrolysis of membrane phospholipids [40] | **1.330^c^** | 0.857 | 7922 |
| **Long-chain fatty-acyl elongase^c^** | ***ELOVL6*** | NM_024090.1 | Long chain fatty acid elongation [43] | **1.570^c^** | 0.885 | 14308 |
| **Cytochrome P450, subfamily IIC, polypeptide 18^c^** | ***CYP2C18*** | NM_000772.1 | Conversion of arachidonic acid to active eicosanoids? [38] | **0.765^c^** | 0.975 | NL |
| **Steroidogenic acute regulatory protein^c^** | ***STAR*** | NM_000349.1 | Cholesterol transport to mitochondria for conversion to steroids [35] | **0.779^c^** | 1.127 | 18238 |

^a^Some non-outlier genes are also listed to provide a global view of expressional changes in cholesterol biosynthesis and regulation.

^b^Multiple transcripts for the same gene in the microarray are listed separately. SD

^c^Outlier genes and the fold-change(s) in their outlier transcript(s) are shown in bold.

NL: gene not listed.
